# Statins and cognition: Modifying factors and possible underlying mechanisms

**DOI:** 10.3389/fnagi.2022.968039

**Published:** 2022-08-15

**Authors:** Tahereh Jamshidnejad-Tosaramandani, Soheila Kashanian, Mohamed H. Al-Sabri, Daniela Kročianová, Laura E. Clemensson, Mélissa Gentreau, Helgi B. Schiöth

**Affiliations:** ^1^Nanobiotechnology Department, Faculty of Innovative Science and Technology, Razi University, Kermanshah, Iran; ^2^Department of Biology, Faculty of Science, Razi University, Kermanshah, Iran; ^3^Department of Surgical Science, Functional Pharmacology and Neuroscience, Uppsala University, Uppsala, Sweden; ^4^Faculty of Chemistry, Sensor and Biosensor Research Center (SBRC), Razi University, Kermanshah, Iran

**Keywords:** statins, Alzheimer’s disease, cognitive function, repurposing, mechanisms, factors

## Abstract

Statins are a class of widely prescribed drugs used to reduce low-density lipoprotein cholesterol (LDL-C) and important to prevent cardiovascular diseases (CVD). Most statin users are older adults with CVD, who are also at high risk of cognitive decline. It has been suggested that statins can alter cognitive performance, although their positive or negative effects are still debated. With more than 200 million people on statin therapy worldwide, it is crucial to understand the reasons behind discrepancies in the results of these studies. Here, we review the effects of statins on cognitive function and their association with different etiologies of dementia, and particularly, Alzheimer’s disease (AD). First, we summarized the main individual and statin-related factors that could modify the cognitive effects of statins. Second, we proposed the underlying mechanisms for the protective and adverse effects of statins on cognitive performance. Finally, we discussed potential causes of discrepancies between studies and suggested approaches to improve future studies assessing the impact of statins on dementia risk and cognitive function.

## Introduction

Dementia prevalence has increased dramatically across the world, currently affecting 50 million people, and it has been reported to be the fifth leading cause of death according to the World Health Organization. With an increase in the aging population, this number is predicted to exceed 130 million by 2050 (Prince et al., 2013). There are many causes of dementia, including Alzheimer’s disease (AD), vascular dementia (VaD) (Emrani et al., 2020), post-stroke dementia, Vascular contributions to Cognitive Impairment and Dementia (VCID) which is a neurocognitive disorder distinct yet overlapping with AD (Montagne et al., 2020). AD is the most common form of dementia (present in 60–70% of the cases) that typically manifests itself through cognitive decline (Silva et al., 2019) and is characterized by cerebral accumulation of amyloid-β (Aβ) plaques, tau neurofibrillary tangles, abnormal neuronal metabolism, subsequent neuronal cell death, and brain atrophy (Bloom, 2014; Picard et al., 2018). Currently, there is no curative treatment for AD due to the combination of complicated etiologies of the disease (Fish et al., 2019). AD is a multifactorial disease caused by a combination of genetic and environmental risk factors (Broce et al., 2019). Pathological factors may play either an accelerating or a leading role in cognitive dysfunction, including neuroinflammation, vascular small vessel disease, and Lewy Body pathology in early 60s (Gauthier et al., 2018). Additionally, hypercholesterolemia at midlife is one of the main risk factors for dementia that can be restricted by using cholesterol-lowering drugs and thus, modifying the incidence of dementia at late-life (Loera-Valencia et al., 2019). Therefore, this could suggest an opportunity for dementia management based on repurposing of existing cholesterol-lowering medications, such as statins (Chew et al., 2020; Sodero and Barrantes, 2020).

Statins are 3-hydroxy-3-methyl-glutaryl-coenzyme A reductase (HMGCR) inhibitors that lower blood cholesterol (Xuan et al., 2020). They are currently the first-line treatment for dyslipidemia in the primary and secondary prevention of major cardiovascular events (Collins et al., 2016). Since the incidence of AD is correlated with dyslipidemia (Loera-Valencia et al., 2019), medications that reduce low-density lipoprotein cholesterol (LDL-C), such as statins, have been suggested as candidate treatments for AD (Zhu et al., 2018; Behl et al., 2020). However, whether statins have beneficial or detrimental effects on cognitive function is still under debate (Fink et al., 2018; Mach et al., 2018; Mejías-Trueba et al., 2018; Ramanan et al., 2018; Kyriakos et al., 2020; Williams et al., 2020; Ying et al., 2021). Nevertheless, the Food and Drug Administration declared reversible minor side effects of memory loss and confusion in the prescribing information for statins in 2012 (Robertson et al., 2019). Paradoxically, recent investigations have revealed that statins can improve cognitive functions such as verbal, working, and logical memory, and reduce the risk of dementia and AD (Schultz et al., 2018; Zhu et al., 2018). In the past, cholesterol levels and statin use effects on AD were reviewed in epidemiological, preclinical (Shepardson et al., 2011a) and human studies (Shepardson et al., 2011b). Shepardson et al. (2011b) showed inconsistent results among studies regarding participants’ age and clinical status. They also discussed the relevance of discrepancies and potential molecular mechanisms regarding the negative effects of cholesterol on the development of AD. In the human studies, they acknowledged some confounding factors including the different abilities of statins to cross the blood-brain barrier (BBB), the stage of AD at which statins were administered, and the pleiotropic metabolic effects of the drugs. Since statin use is crucial for primary and secondary prevention of CVD and the incidence of cognitive decline is higher in older adults, an up-to-date and comprehensive review addressing the effects of statins on cognitive function considering all possible confounding factors was needed. A review of 24 recent studies on the cognitive effects of statins has shown that the results of previous studies were contradictory. Among 1,404,459 participants over 60 years old, no significant association was found between statin use and all-cause dementia (Adhikari et al., 2021). Petek et al. (2018) and Yang et al. (2020) have summarized recent information on the statin cognitive effects in relation to the renin-angiotensin metabolism, blood pressure, and brain cholesterol, as well as the mechanisms by which statins can influence neurodegeneration (Petek et al., 2018; Yang and Williamson, 2019). Moreover, Schultz et al. (2018) have suggested independent mechanisms for protective and harmful cognitive effects of statins that are modified by ethnicity, sex, the properties of the statin molecule, and genetic and biological factors (e.g., decreased drug metabolism) (Schultz et al., 2018). Additionally, Kim et al. (2020) have considered sex in the subgroups of statin types, exposure duration, and patients’ age. They have suggested that sex is a modifying factor on the statin cognitive effects in people with ischemic heart disease (IHD) (Kim et al., 2020). In contrast, Petek et al. (2018) have highlighted the opposite results regarding the effects of statins on memory and neurodegeneration and attributed it to the variability in inherent characteristics of statins, such as the ability to penetrate the BBB, genetic factors, and cholesterol-dependent and independent (pleiotropic) effects of statins on cognitive function (Petek et al., 2018). Finally, Wełniak et al. (2020) have discussed the potential of statin therapy on dementia by exploring how statins affect characteristic features of AD pathophysiology such as Aβ, tau protein, brain cholesterol metabolism, and cognitive assessment differences (Wełniak et al., 2020).

**FIGURE 1:**
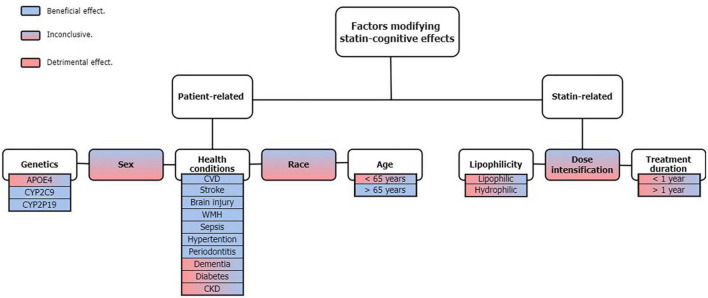
Classification of recognized factors that can modify the statin effect on cognitive function. APOE4, apolipoprotein E isoform 4; CYP2C9, cytochrome P450 family 2 subfamily C member 9; CYP2P19, cytochrome P450 2 subfamily C member 19; CVD, cardio vascular disease; WMH, white matter hyperintensities; CKD, chronic kidney disease.

In this review, we explored the individual-related factors, such as genetic factors, sex, ethnicity, age, and comorbidities, as well as statin-related ones like lipophilicity, dosage, and the duration of treatment (Figure 1). Then, we discussed the possible underlying mechanisms for beneficial and detrimental effects of statins on cognitive performance, explain the likely underlying reasons for discrepancies between studies and, suggest guidelines for future studies.

### Approach

The PubMed database was searched for reviews, systematic reviews, meta-analyses, and original research articles covering 2018–2021 dates with the keywords “Alzheimer’s disease [MeSH]” OR “cognitive function [MeSH],” AND “statins.” Articles were retrieved and reviewed to objectively evaluate the effects of statins on cognitive function, the risk of AD, the modifying factors, and the underlying mechanisms.

## Individual-related factors

The effect of statins on cognitive function differs according to individual-related factors such as their genetic diversity, ethnicity, sex, age, and comorbidities. This may affect the practitioners’ as well as patients’ decisions in using statins for CVD treatment. With the increase in life expectancy, the prescription of statins is likely to rise in the next few years. Therefore, the association between statin use and cognitive function according to the individual-related factors should be clarified.

### Genetic factors

Genetic factors play a pivotal role in AD incidence (Carmona et al., 2018). The apolipoprotein E ε4 allele (*APOE4*) is the strongest genetic risk factor for AD (Liao et al., 2017). ApoE is a key extracellular protein, involved in various functions such as lipid transport, cholesterol homeostasis, synaptic plasticity, degeneration of brain capillary pericytes, and BBB dysfunction but, its exact role in cognitive function is still elusive (Belloy et al., 2019; Safieh et al., 2019; Montagne et al., 2020). *APOE4* can contribute to AD risk through its impact on various brain cell types, such as astrocytes and microglia (Julia et al., 2022). *In vitro* and *in vivo* studies showed intracellular cholesterol accumulation in *APOE4* human astrocytes (Julia et al., 2022). Additionally, *in vitro* studies on isogenic astrocytes derived from iPSC (induced Pluripotent Stem Cells) have shown that *ApoE2*, *E3* and *E4* differentially modulate cellular homeostasis, cholesterol metabolism, and inflammatory response (de Leeuw et al., 2022). Since 25% of the population is *APOE4* carrier, it is important to understand its role in AD pathology and its impact on the association between statin use and cognitive function. However, the correlation between *APOE4* carrier status and the cognitive benefits of statins is poorly understood. Recently, a meta-analysis showed that statins have more cognitive benefits in *APOE4* carriers and patients with higher cholesterol levels (Xuan et al., 2020). Similarly, in *APOE4* carriers, a longitudinal study has found that statin use was associated with a slower rate of global cognition decline over 6 years compared with non-users in community-dwelling elderly Australians age 70–90 years. Meanwhile, in non-*APOE4* carriers, the rate of memory or cognitive decline in long-delayed recall performance was similar between statin users and non-users (Samaras et al., 2019). Also, it has been reported that while the users of statins showed an increased risk of AD, only *APOE4*-carrier statin users displayed a slightly lower risk for AD and dementia, especially in men (Dagliati et al., 2020). Nevertheless, autopsy evidence of statin users in autopsy-confirmed Alzheimer’s dementia brains did not demonstrate a significant difference in any AD pathological neuroimaging markers, suggesting that the statin use neither improves nor worsens AD pathology according to their *APOE4* status (Crum et al., 2018).

Several studies have indicated that single nucleotide polymorphisms (SNPs) can alter the pharmacokinetics of statins (Smiderle et al., 2016; Naito et al., 2017; Ahangari et al., 2020). For example, the genetic variant of the *cytochrome P450* (*CYP*) family genes, encoding for the main enzymes of the hepatic metabolism of statins, can alter body exposure to statins, making patients more vulnerable to their effect on cognitive function (Schultz et al., 2018). It has been reported that the *CYP2C19* polymorphism (rs4388808) confers protection against the Aβ burden in AD patients (Benedet et al., 2018). Besides, the gene encoding for *CYP2C9* is linked to familial AD. CYP enzymes are also responsible for the metabolism of certain antihypertensive drugs, which are most often concomitantly used in older adults (Barthold et al., 2020), suggesting that some combinations of statins and antihypertensive drugs may alter the activity of CYP enzyme family and subsequently have a various effect on Alzheimer’s disease and related dementias (ADRD) risk. Consequently, the inconsistency among studies regarding the effects of statins on cognitive performance or memory impairment could be explained by differences in drug metabolism and transport (i.e., pharmacokinetic interactions between certain statins with other drugs and certain genetic variations of CYP enzymes) (Barthold et al., 2020). The effect of CYP polymorphisms on the cognitive effects of statins should be considered in future clinical trials as they can mask the outcomes of the analysis.

Moreover, the HMGCR gene polymorphisms can influence both the cholesterol-lowering response to statin and the pleiotropic statin protective effect on cognitive function (de Oliveira et al., 2022). It has been reported that rs3846662 might increase the HMGCR expression and thereby contribute to the onset and progression of AD (Ma et al., 2019). Conversely, rs17238484 was associated with a minor reduction in the risk of AD (Hon-Cheong et al., 2017). Additionally, a study conducted on three cohorts evaluated the association between AD and the *HMGCR’s* rs3846662 G negative status and highlighted that this variant was one of the most important protective genetic factors for AD, behind *APOE2* (Leduc et al., 2015). However, Mendelian randomization analyses led on HMGCR did not suggest that the use of statins could alter AD risk (Williams et al., 2020). Finally, a pilot study showed that the genetic variants of CETP (Cholesteryl Ester Transfer Protein), rs5882-AA, and, the genetic variant of NR1H2 (Nuclear Receptor subfamily 1 group H member 2), rs2695121-CC, were associated with cognitive dysfunction, especially in patients using lipophilic statins. However, the effect of rs3846662 (HMGCR variant) had not been able to be evaluated (de Oliveira et al., 2022). More studies are needed to elucidate the exact effect of HMGCR gene polymorphisms in statin users.

### Ethnicity

Ethnic differences influence the efficacy of statins in lowering LDL-C (Naito et al., 2017). A 7-year follow-up study demonstrated that ethnic and sex differences influenced AD risk. For instance, simvastatin was associated with lower AD risk for white men and women, Hispanic men and women, and black women, whereas pravastatin and rosuvastatin were associated with reduced AD risk for white women (Zissimopoulos et al., 2017). Recently, a combination of different statin types and different classes of antihypertensive drugs have shown ethnicity-dependent beneficial effects on dementia risk. Among white participants, combined use of angiotensin receptor blockers (ARBs) and any type of statin was associated with a lower risk of AD whereas no significant association was found among black participants using rosuvastatin or among Hispanic participants (Barthold et al., 2020).

### Sex

It is well known that sex modifies the effect of statins on cognition. Recently, the association between statins and the risk of dementia was examined in individuals with hypercholesterolemia over an 11-year follow-up. For both men and women, statin use was associated with a more than 35% decrease in the risk of all-cause dementia and a protective effect of statins on vascular dementia was only found significant in women (Lee J. W. et al., 2020). Another study found that lovastatin reduces the risk of dementia in women and that atorvastatin decreases the risk of dementia in men (Kim et al., 2020). Unlikely, men statin users showed significantly faster logical memory decline than non-users, while the results were comparable in women (Samaras et al., 2019). These conflicting results reveal the importance of sex differences in the association between the statin effects and the risk of dementia.

Such sex differences can be influenced by genetic factors. For example, it has been shown that *APOE4* carrier status has fewer benefits on cognitive function in women than in men statin users (Dagliati et al., 2020). Moreover, clinical data have shown that women tend to metabolize medications faster than men, especially substrates of CYP3A4 (Faubion et al., 2019). Thus, variability in the genetic coding for drug-metabolizing enzymes and transporters may also underlie the sex differences. Simvastatin, lovastatin, and atorvastatin are metabolized in the liver by *CYP3A4/5* and fluvastatin is metabolized primarily by *CYP2C9*. These enzymes have shown significant inter-individual variable inactivity as well as induction and inhibition mediated by endogenous and exogenous substances due to genetic variation. However, it remains unclear whether an interaction between genetic variants and sex could impact statin response and adverse cognitive effects (Faubion et al., 2019).

**FIGURE 2:**
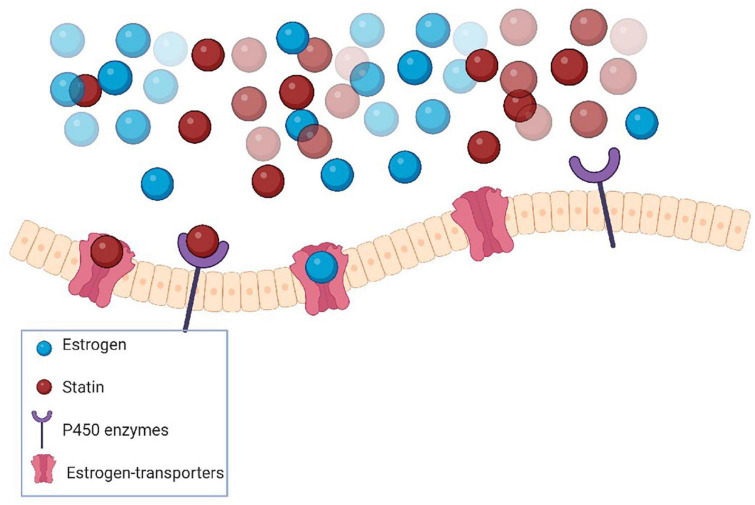
Statins-estrogen hormone interactions are probable because of the common metabolic pathways in the liver with the same cytochrome P450 enzymes. Also, statins could compete with estrogens for the same transporters.

The reason for sex differences in the effect of statins on AD and cognitive performance is still not fully elucidated. However, statin interaction with estrogen receptors could offer a plausible explanation. Estrogen receptors, encoded by *ESR genes*, play a critical role in regulating cellular metabolism and bioenergetics in metabolically active tissues such as muscle, kidney, heart, and brain (Lynch et al., 2018). Statins have been shown not only to reduce (Hohl and Roman, 2004) and inhibit estrogen receptors (Alisha and Tripti, 2021) but also, potentially interact with estrogen receptors in multiple body systems (Faubion et al., 2019; Figure 2). Additionally, hormonal phases of women might influence statin efficacy through competition with estrogen for binding sites and regulatory pathways affected by statins or genetic variants in enzymatic targets for statins (Faubion et al., 2019). Despite the importance of estrogen in memory function, AD prevention (Uddin et al., 2020), and the change of estrogen levels in aging and health conditions, no studies have considered the possible interaction of statins and estrogen in their analysis (Faubion et al., 2019). Thus, it could be interesting to scrutinize the statin effect on cognitive function in both sexes in relation to estrogen levels, taking into consideration the treatment duration, age, and relevant estrogen-related health conditions.

### Age

Aging is the major risk factor for memory loss and other diseases that influence cognitive function like AD, depression, sleep disorder, CVD, symptom-free intracranial atherosclerotic stenosis, and diabetes (Nanna et al., 2018; Shetty et al., 2019; de Almeida et al., 2020; Orock et al., 2020; Viscogliosi et al., 2020). Statins have been shown to improve age-related conditions like CVD and AD (Chu et al., 2018; Newman et al., 2019; Shetty et al., 2019; van Dongen et al., 2019). Generally, after a diagnosis of dementia, physicians reassess the medications used by patients (Picton et al., 2021). Thus, the findings could be attributed to reverse causation and confounding factors (Power et al., 2015). However, a recent evaluation of the evidence for the long-term and short-term effects of statin therapy in patients with dementia older than 65 has indicated a small benefit in delaying the progression of AD (Davis et al., 2020). The evidence was insufficient to fully evaluate the effect of statins and their dosage on cognitive function in people who already have dementia. Another study has evaluated the effect of age on the cognitive function of statin users and non-users. This study compared 900 statin users and 5000 non-users from the UK Biobank. Results showed that the effects of statins vary significantly with age. Indeed, individuals over 65 showed an improvement in reaction time whereas individuals under 65 displayed impairment in working memory. The same study has shown both age groups of statin users benefited from statin treatment, but the benefit was stronger in individuals under 65, suggesting that statin therapy in midlife could be more beneficial for cognitive function (Alsehli et al., 2020). The modifying effect of age on the association between statin and AD has been supported by other studies including statin types and sex covariates. A nationwide study including three age categories, 65–75, 76–85, and 86 with IHD showed inconsistent results, depending on the type of statins. In the age subgroup of 65–75, rosuvastatin and pravastatin use were associated with a reduced risk of AD. In the age subgroup of 75–85, atorvastatin and rosuvastatin use was associated with reduced AD risk. However, no significant association was found in the age subgroup of 86 and older, which could be explained by the lower sample size in this subgroup (Kim et al., 2020). Yet, a recent study has found that the association between statin therapy and cognitive function is not modified by age (Roy et al., 2020). Likely, in a nationwide population-based cohort study, statin use was associated with a lower risk of dementia, but no meaningful effect for the relationship between statin use and the risk of dementia was found for age (Pan et al., 2018). Although this discrepancy might be due to the limited sample size, further age-specific investigations are needed to better understand the effects of statins on cognitive function in relation to age.

Age modifying effects on the cognitive benefits of statins have been explained by Volloch et al. (2020), suggesting that both sporadic and familial AD regardless of their origin, have two definite stages. The first slow stage of intracellular Aβ accumulation causes insignificant damage and also occurs in healthy individuals. The second fast stage starts shortly before the onset of AD symptoms. During this stage, levels of Aβ increase sharply causing significant damage and resulting in AD symptoms. This study recommended statin therapy as a preventive treatment for dementia and claimed that statin therapy should be initiated before the onset of the second stage to be more effective (Volloch et al., 2020). Recent studies have confirmed this age (or stage) dependent effect of statins on cognitive function (Chu et al., 2018; Chadha and Frishman, 2020). Furthermore, several other possible direct and indirect pathways in midlife can also modify cognitive function later in life, like cerebral lipid content, higher fasting triglycerides levels in circulation, vascular risk factors (Larsson and Markus, 2018; Nägga et al., 2018; Cheng et al., 2020; Dai et al., 2021), the connection between lipid quantities and Aβ pathology (Nägga et al., 2018), chronic systemic inflammatory state and neuroinflammation with aging (Calsolaro and Edison, 2016; Chung et al., 2019). Future studies are recommended to decipher the statin cognitive effect in regard to the aging process.

### Comorbidities

The existence of comorbidities may also explain the inconsistent results of studies on the cognitive effects of statins. Thus, it is important to consider different health conditions while assessing the cognitive effects of statins (Table 1).

**TABLE 1:** Different health conditions of patients and their impacts on the statins’ cognitive effects.

Health conditions	The effect of statins on cognitive function	References
Hypertension	The use of pravastatin and rosuvastatin in combination with AHTs was effective at reducing the risk of dementia. But, long-term candesartan plus hydrochlorothiazide, rosuvastatin, or their combination showed no meaningful effect on cognitive function.	Bosch et al., 2019; Barthold et al., 2020
CVD	Statins can modify the risk of dementia by reducing vascular risk factors such as LDL-C levels, infarcts risk, white matter lesions, and cerebral microbleeds. The use of statins can lower dementia risk in older IHD patients.	Pal et al., 2018; Offer et al., 2019; Kim et al., 2020
Diabetes	The harmful effect of statins on cognitive aging related to T2D was small in comparison with the expected benefits on cerebrovascular events in the HPS assessment. Intensification of statin therapy increases the frequency association of T2D among patients with cognitive impairment.	Offer et al., 2019; Roy et al., 2020
WMH	Statin therapy was associated with a lower risk of WMH and cognitive impairment.	Zhang et al., 2019; Cheng et al., 2020
Periodontitis	The use of statins in individuals with periodontitis was associated with a reduced risk of dementia.	Lee C. Y. et al., 2020
Sepsis	Statin therapy resulted in an anti-inflammatory effect in the sepsis brain and reduced cognitive deficits.	Catalão et al., 2017; Tian et al., 2019; Tauber et al., 2020

AHT, anti-hypertensive treatment; CVD, cardiovascular disease; HPS, heart protection study; IHD, ischemic heart disease; LDL-C, low-density lipoprotein cholesterol; T2D, type 2 diabetes; WMH, white matter hyperintensity.

#### Hypertension

Hypertension is a modifiable risk factor for dementia (Walker et al., 2019; Yang and Williamson, 2019). Approximately 25% of adults over the age of 65 use both anti-hypertensive treatments (AHTs) and statins, which were independently associated with lower dementia risk (Barthold et al., 2020). Recently, Barthold et al. (2020) compared dementia risk associated with concurrent use of different combinations of statins and AHTs. The use of pravastatin and rosuvastatin in combination with renin-angiotensin system (RAS)-acting AHTs significantly reduced the risk of dementia (Barthold et al., 2020). However, long-term use of candesartan with hydrochlorothiazide, rosuvastatin, or their combination to lower blood pressure demonstrated no significant effect on cognitive function in participants without known CVD or need for treatment (Bosch et al., 2019).

#### Cardiovascular diseases

Statins may have cognitive benefits through lowering other risk factors implicated in CVD, such as pathogenic vascular changes in the brain (infarcts, white matter lesions, and cerebral microbleeds) that could increase the risk of cognitive decline with age (Moroni et al., 2020). In the Heart Protection Study (HPS), statin use for 5 years prevented 2.0% of patients from a non-fatal stroke or transient ischemic attack and 2.4% from a non-fatal cardiac event, which led to an expected reduction in cognitive aging of 0.15 years (Offer et al., 2019). Since IHD is an established risk factor for dementia, anti-hyperlipidemia, anti-platelet, and anti-hypertensive therapy have been suggested to reduce dementia risk. A study has reported the association between the use of different statin types and the risk of dementia stratified by sex. Older women with a history of IHD displayed a fivefold increase in the risk of dementia (Kim et al., 2020).

#### Chronic kidney disease

Chronic kidney disease (CKD) increases the risk of dementia, damages the cerebrovascular system, and promotes WMH in the prefrontal cortex in animal models (Viggiano et al., 2020). The beneficial effects of statin therapy for CVD in patients with CKD remain controversial, and the cognitive effect of statins is rather unknown in the CKD population (Markossian et al., 2019). Recently, Zijlstra et al. (2020) investigated the association between kidney function and cognitive decline in older adults at high risk of CVD, using data from the Prospective Study of Pravastatin in the Elderly at Risk (PROSPER). For all cognitive tests, patients with CKD at stage 4 had the poorest scores at the baseline. A trend for faster cognitive decline over time regardless of higher risk for stroke and the transient ischemic attack was reported. However, this was not reported for CKD at stages 3a and b. Additionally, the correlation between severe kidney failure and cognitive decline was more significant in patients with a history of CVD, possibly due to a higher likelihood of vascular damage in the kidney and brain (Zijlstra et al., 2020). Thus, by lowering the risk of CVD, statin therapy might lower the risk of CKD and thus, future studies in this regard are warranted.

#### Diabetes

Type 2 diabetes (T2D) is known to exacerbate AD (Chatterjee and Mudher, 2018). Beside, T2D is characterized by insulin resistance which contributes not only to hyperglycemia but also to hyperlipidemia, inflammation, oxidative stress, and atherosclerosis (Arnold et al., 2018). Therefore, understanding the effects of statins on both T2D and AD might help repurpose statins for AD. In the HPS, statin therapy modestly increased the risk of T2D (Offer et al., 2019). Besides, the intensification of statin therapy may alter the effect of diabetes on cognition. The association frequency of T2D was significantly higher in the patients on High-Intensity Statin Therapy (HIST) with cognitive impairment in comparison with patients without cognitive impairment. There was no difference in the frequency of association of T2D in the patients on Moderate-Intensity Statin Therapy (MIST) with or without cognitive impairment (Roy et al., 2020). Nevertheless, it has been suggested that the benefits of statin use in the prevention and treatment of CVD outweigh the possible adverse effects of statins on cognitive aging associated with T2D (Offer et al., 2019). Further studies should evaluate whether the beneficial effect of statins on cognition does not come at the expense of an increased risk of T2D.

#### White matter hyperintensities

Cerebral white matter hyperintensities (WMH) and cognitive impairment are common in hypertensive older adults (Calsolaro and Edison, 2016). Two randomized, double-blind, and placebo-controlled clinical trials for newly diagnosed mild cognitive impairment (MCI) subjects and older patients undergoing antihypertensive treatment showed that statin therapy was associated with less WMH (Zhang et al., 2019; Cheng et al., 2020). Furthermore, rosuvastatin use was associated with a lower risk of cognitive impairment and the combination of rosuvastatin with telmisartan may reduce WMH progression by maintaining the integrity of cognitive function in older patients with hypertension (Zhang et al., 2019).

#### Stroke

The association between stroke and dementia risk is well established (Bunch et al., 2020). Statin use is correlated with a notably lower incidence of post-stroke dementia. Interestingly, a better efficiency was found when the duration of statin therapy was longer and when patients were treated with lipophilic and high-potency statins (Pan et al., 2018). Consistent with this study, a meta-analysis has shown that atherosclerosis was associated with a higher risk of post-stroke dementia. Besides, post-stroke use of statins lowered the risk of cognitive impairment, independently of age (Yang et al., 2020). Nevertheless, statins may increase the risk of hemorrhagic stroke in patients who have already had a stroke (Adhyaru and Jacobson, 2018).

#### Brain injury

Statin therapy has been reported to decrease the risk of ADRD in individuals with brain injury. A study has demonstrated that statin use was associated with a reduced risk of stroke, depression, AD-related dementia, and mortality following traumatic brain injury (TBI) (Khokhar et al., 2018). Another study has indicated that the combination of statins and angiotensin-converting enzyme (ACE) inhibitors significantly lowered the risk of dementia with possible AD in a cohort of people with a history of TBI (Li M. et al., 2020). Additionally, statin therapy modestly lowered the risk of subsequent dementia in adults after a concussion (Redelmeier et al., 2019). Overall, these findings emphasize the importance of statin use for improving cognitive function in patients with a history of brain injury.

#### Periodontitis

A recent nationwide retrospective cohort study has highlighted that periodontitis was associated with higher dementia risk. Notably, the association between periodontitis and dementia risk was significant in people older than 60. Among individuals with periodontitis, the use of statins was associated with a reduced risk of dementia, while as expected, diabetes, mental disorders, and stroke were the major risk factors for dementia (Lee C. Y. et al., 2020).

#### Sepsis

Sepsis-associated encephalopathy and septic encephalitis were both reported to be associated with long-term cognitive impairment. Since sepsis is a fatal systemic inflammatory response to infection, drugs that possess anti-inflammatory properties like statins may be effective. Tauber et al. (2020) have suggested a possible neuroprotective effect of simvastatin in the brain structures involved in spatial learning and memory and raised the need for behavioral studies evaluating the impact of sepsis on cognitive damage (Tauber et al., 2020). In animal studies, therapy with either simvastatin or atorvastatin resulted in an anti-inflammatory effect in the sepsis brain and reduced cognitive deficits (Catalão et al., 2017; Tian et al., 2019). Moreover, an epidemiological study demonstrated that simvastatin and atorvastatin were associated with a lower risk of mortality whereas rosuvastatin was not. These results suggest that the effect of statins on sepsis is not correlated to their lipid-lowering potency (Lee et al., 2018). However, in a retrospective cohort study, no association was found between statin use and sepsis risk in patients with dementia (Yeh et al., 2019). Moreover, the protective effect of statins on sepsis in clinical trials has yielded conflicting results which may be explained by different treatment duration and dosages. Besides, the effect of statins on sepsis is limited to the early phases of sepsis (Eladwy and Bazan, 2020). A neuroprotective effect of statins on sepsis might be caused by the modulation of leukocyte and monocyte functions, the production of proinflammatory cytokines, improvement in endothelial function, reduction of oxidative stress, and platelet activity (Eladwy and Bazan, 2020).

Collectively, the effect of statins on cognitive function and AD incidence varies widely depending on individual-related factors such as their genetic factors, ethnicity, sex, age, and comorbidities. Among these, aging is the main risk factor for memory loss and other diseases that influence cognitive function. Additionally, attention to the incidence of different AD may highlight the cognitive effects of statins. Therefore, it is important to consider all the individual-related factors when assessing the statin cognitive effects.

## Statin-related factors

Lipophilicity, dosage, and treatment duration of different types of statins might alter their effects on cognitive function. Studying these statin-related factors in addition to the individual-related factors may expand our understanding of the effect of statins on cognitive function.

### Statin lipophilicity

The inherent properties of statins, like lipophilicity, might impact their effects on cognitive function. So far, the effects of statin lipophilicity on cognitive function are inconclusive (Table 2).

**TABLE 2:** The statins lipophilicity and their effects on cognitive function.

Statins solubility	Statins type	Effects on cognitive function
Lipophilic	Atorvastatin	Significantly decreased dementia risk in patients with stroke (Pan et al., 2018). Major reduction in the dementia risk (Poly et al., 2020). Reduction of dementia risk only in men with IHD (Kim et al., 2020). Preclinical meta-analysis showed the maximum reduction of Aβ plaques (Rahman et al., 2020).
	Lovastatin (Fungal)	Not associated with reduced risk of dementia (Poly et al., 2020). Significant reduced risk of dementia in women with IHD (Kim et al., 2020). Preclinical meta-analysis failed to demonstrate any protection against Aβ plaque load (Rahman et al., 2020).
	Fluvastatin	Significantly decreased dementia risk in patients with stroke (Pan et al., 2018). Not associated with reduced risk of dementia (Poly et al., 2020).
	Simvastatin	Significantly decreased dementia risk in patients with stroke (Pan et al., 2018). Presented a higher AD risk (Sinyavskaya et al., 2018). Large reduction in dementia risk (Poly et al., 2020). No significant reduced risk of dementia in patients with IHD (Kim et al., 2020). Preclinical meta-analysis showed a reduction of Aβ plaques (Rahman et al., 2020). Long-term subcutaneous treatments of simvastatin damaged mouse hippocampal synaptic plasticity, and therefore, recognition, and spatial memory (Guo et al., 2021).
	Pitavastatin	Preclinical meta-analysis showed a reduction of Aβ plaque (Rahman et al., 2020).
Hydrophilic	Rosuvastatin	Significantly decreased dementia risk in patients with stroke (Pan et al., 2018). In combination with AHTs, reduced the risk of AD and related dementia (Barthold et al., 2020). Major reduction in dementia risk (Poly et al., 2020). Associated with the greatest (18%) protective effect on dementia risk in patients with IHD (Kim et al., 2020).
	Pravastatin (Fungal)	In combination with AHTs, reduced the risk of AD and related dementia relative (Barthold et al., 2020). Not associated with reduced risk of dementia (Poly et al., 2020). Reduction of dementia risk in patients with IHD (Kim et al., 2020).

Aβ, amyloid-β; AD, Alzheimer’s disease; AHT, anti-hypertensive treatment; IHD, ischemic heart disease.

Lipophilic statins are thought to have greater impacts on cognitive function (Ward et al., 2019; Chadha and Frishman, 2020; Samant and Gupta, 2020). For example, Pan et al. (2018) have reported higher benefits for lipophilic statins in AD risk reduction. On the contrary, lipophilic statins have been associated with higher AD risk compared to hydrophilic statins in another study (Sinyavskaya et al., 2018). This might be explained by the greater general ability of lipophilic statins to cross the BBB by passive diffusion across cell membranes, and act more easily in other tissues such as the brain, adipose tissue, and muscle (Sodero and Barrantes, 2020). It should be noted that factors modifying cholesterol levels, i.e., biosynthesis, elimination, and turnover in the brain are important in AD management (Loera-Valencia et al., 2019). The different effects of statin on cognitive functions may be the result of low brain cholesterol levels, regardless of statin type (Ward et al., 2019; Chadha and Frishman, 2020).

A recent systematic review indicating that hydrophilic statins had more protective effects in preventing all-cause dementia and possibly AD in comparison to lipophilic statins (Chu et al., 2018). Consistently, a meta-analysis has shown that hydrophilic statins lowered the risk of dementia more strongly (28%) than lipophilic statins (16%) (Poly et al., 2020). Generally, hydrophilic statins need a carrier protein to be transported to tissues other than the liver (Sodero and Barrantes, 2020). The difficulty in explaining the influence of statin lipophilicity on cognition can be ascribed to the diverse effects of statins on different types of dementia based on their lipophilicity. For example, a meta-analysis of observational studies by Poly et al. (2020) has reported that hydrophilic statins were associated with a lower risk of all-cause dementia, and lipophilic statins were associated with a lower risk of AD, but not vascular dementia (Poly et al., 2020). Additionally, increased adipose tissue and food intake may change the absorption, bioavailability, and distribution of statins due to their lipophilicity (Gheorghe et al., 2020).

Nevertheless, several studies reported the effects of statins on cognitive function regardless of statins lipophilicity (Ramanan et al., 2018; Samaras et al., 2019). A study on the cumulative effects of long-term statin treatment in five lipophilicity groups has highlighted that the beneficial effects of statins on cognitive function were independent of their lipophilicity. Additionally, the same study has demonstrated dissimilar effects for each type of statins in reducing the risk of AD (Kim et al., 2020). It should be mentioned that hydrophilic statins were used less frequently and thereby the data for each type of these drugs were insignificant. Furthermore, the estimates were often unreliable due to the limited sample size (Zissimopoulos et al., 2017). Discrepancies among the results of the studies concerning the effects of statins according to their lipophilicity are presented in Table 2.

### Dosage

Studies have demonstrated that the pharmacological effects of statins are different according to the dosage (Naito et al., 2017; Nanna et al., 2018). Although several studies have evaluated the impact of statins’ dosage on cognitive function, it is difficult to compare studies because the dosage was reported differently. Additionally, some studies demonstrated that the effects of statins on cognitive function were dose-dependent but other studies showed the opposite (Table 3). For instance, Zhang et al. (2019) showed that low-dose statins (10 mg per day) did not drastically reduce blood lipids drastically but significantly decreased the risk of cognitive decline (Zhang et al., 2019). The effects of statins of AD risk are complicated and not limited to the dose.

**TABLE 3:** The effect of statins dose intensification on cognitive function.

Dose-dependent effects on cognitive function	Protective	A meta-analysis showed that a 5-mg increase in the daily dose of statins was correlated with an 11% decrease in dementia risk (Zhang et al., 2018).
		A cohort study showed higher protective effects for a high dose (the cumulative defined daily doses ranged from 28 to 365, 366 to 730, and more than 730) of statin than for a lower dose (Chang et al., 2019).
		A randomized controlled trial on older adults with hypertension showed that long-term use of 10 mg rosuvastatin may reduce WMH progression and cognitive impairment (Zhang et al., 2019).*
	Harmful	HIST (atorvastatin 40–80 mg, rosuvastatin 20–40 mg) was associated with a higher frequency of cognitive impairment compared to MIST (atorvastatin 10–20 mg, rosuvastatin 10 mg, simvastatin 20–40 mg, and pravastatin 40–80 mg) (Roy et al., 2020).
Dose independent effects on cognitive function	Protective	The statins use was negatively associated with all-cause dementia and vascular dementia in older adults regardless of statins dosage (10–80 mg per day) and LDL-C level (Zingel et al., 2021).
	No harmful	Statin switching from low-dose simvastatin (≤20 mg/day) to high-dose atorvastatin (40 mg/day) did not significantly worsen cognitive function, in patients with T2D (Thongtang et al., 2020).

*A synergistic interaction is reported between telmisartan and low-dose rosuvastatin, as an effective management strategy for the development and progression of WMH and cognitive impairment (Zhang et al., 2019). HIST, high-intensity statin therapy; LDL-C, low-density lipoprotein cholesterol; MIST, moderate-intensity statin therapy; T2D, type 2 diabetes.

### Treatment duration

The effects of statin therapy on cognitive function can be modulated by treatment duration and age (Alsehli et al., 2020). Among patients with stroke, the duration of statin use was associated with a decreased dementia risk, independently of age. Patients using statins for more than 1 year had a significantly reduced risk of dementia compared with non-users. In contrast, statin use for less than 1 year had no protective effects on the incidence of dementia (Pan et al., 2018). Consistently, evidence from a preclinical meta-analysis found that the duration of statin therapy was a covariant in the effects of statins on cognitive function. Pooled estimates of the studies in which statins were administered for > 6 months showed a maximum effect on cognition with low heterogeneity compared with studies in which statins were used for less than 1 month (Rahman et al., 2020). However, the minimum duration required for statins to have an effect on cognitive function in AD patients remains controversial (Ramanan et al., 2018). Moreover, it has been shown that the duration of statin therapy is interdependent with patients’ age (Zhang et al., 2018).

Furthermore, study results on the effect of statin treatment duration on cognitive function vary considerably according to the type of statins. Among older patients with IHD, statin therapy for more than 1 year tended to be associated with a lower risk of dementia for most of the statins type (i.e., atorvastatin, rosuvastatin, pitavastatin, pravastatin, and fluvastatin). However, the risk of dementia was reported to be raised for less than 1 year of use of atorvastatin and simvastatin. Besides, men using atorvastatin, rosuvastatin, or pravastatin for more than 1 year were less likely to develop dementia, and women using atorvastatin, rosuvastatin, pitavastatin, or lovastatin for more than 1 year appeared to have a reduced dementia risk. However, therapy for less than 1 year with either atorvastatin or simvastatin was associated with an increased risk of dementia in women only. Therefore, in addition to the treatment duration, the study results depended on sex, age, and type of statins used (Kim et al., 2020). Finally, in patients with AD, statin therapy appears to improve global cognition in short term (≤12 months), as measured by the Mini-Mental State Examination (MMSE), but no significant improvement was found in the longer time (Xuan et al., 2020).

Statins can modulate cognitive function according to their inherent properties such as lipophilicity, dosage, and treatment duration. Yet, most of these independent studies are inconclusive. Taken together, in addition to the aforementioned individual-related factors, statin-related factors should be considered in future research concerning the effects of statins on AD and cognitive effects.

## Underlying mechanisms for statins’ effects on cognitive function

**FIGURE 3:**
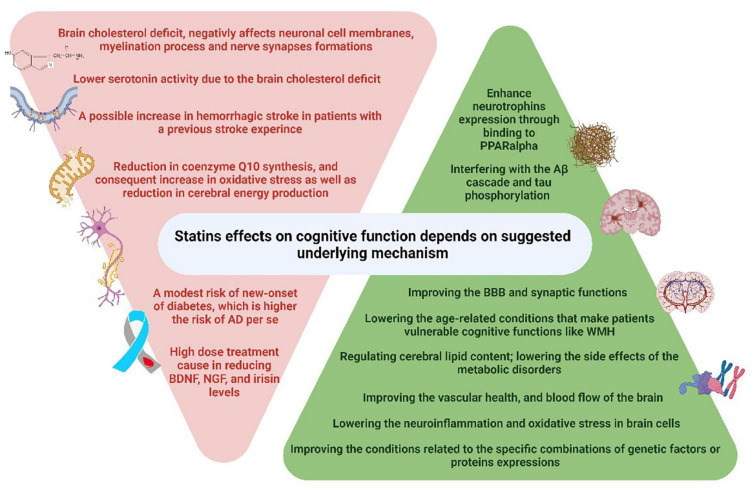
Underlying mechanisms for statins’ detrimental and beneficial effects on cognitive function.

The exact mechanism by which statins influence cognition is still unknown. The underlying mechanisms of the effect of statins on cognitive function are currently being investigated from different perspectives (Figure 3). There is evidence for and against the hypotheses of cholesterol-dependent and –independent effects of statins, anti-inflammatory and antioxidant effects of statins, as well as modulation of transcriptional activities by statins.

### Cholesterol-dependent and –independent effects of statins

Statins may have cholesterol-dependent and –independent (pleiotropic) effects on cognitive function (Petek et al., 2018). Still, the exact underlying mechanisms of the beneficial effects of statins on AD are unknown, but they have been shown to modify several AD-related features (Samant and Gupta, 2020). The cellular and molecular AD-related features that can be modified by statin treatment are: Aβ cascade (Shakour et al., 2019; Behl et al., 2020), tau phosphorylation (Blanchard and Tsai, 2019), oxidative stress, apoptosis and neuroinflammation (Bagheri et al., 2020), BBB function (Li et al., 2018; Lu et al., 2018; Christophe et al., 2020), neurotransmission, synaptic function and plasticity (Forrest et al., 2018; Bukiya et al., 2019), and susceptibility to metabolic disorders (Pal et al., 2018). Moreover, other dementia-related factors can be altered by statins such as the cerebral lipid content and metabolism (Petek et al., 2018; Borroni et al., 2020; Sodero and Barrantes, 2020; Dai et al., 2021), cerebral vascular health and blood flow (Li et al., 2018; Offer et al., 2019; Yang and Williamson, 2019), and white matter integrity (Zhang et al., 2019). Statins reduce the coenzyme Q10 synthesis, resulting in a consequent increase in oxidative stress, and a reduction in cerebral energy production (Tan et al., 2019). Furthermore, inhibition of cholesterol synthesis depletes the central nervous system of myelin (Bagheri et al., 2020) and reduces cholesterol availability to neurons. This contributes to lower serotonin activity, which can cause behavior changes that are detrimental to cognitive function (Solmaz et al., 2020). The effects of statins on the different cellular and molecular mechanisms involved in cognitive impairment are an elusive subject and the study results need to be better recognized, which requires further clinical and animal studies (Ong et al., 2018; Ramanan et al., 2018).

All the methods that target excess cholesterol in the brain, may be important in AD management, depending on the major mechanisms of cholesterol biosynthesis, elimination, and turnover (Loera-Valencia et al., 2019; Petrov and Pikuleva, 2019). However, cholesterol-lowering therapies showed different effects on cognitive function in AD patients. The relationship between cholesterol metabolism in the brain, and peripheral circulation, and whether they should be studied independently or together in AD patients remains unclear. Future studies addressing this confusion are highly suggested.

It has been shown that statins can decrease the levels of brain oxygenated derivatives of cholesterol, and oxysterols (Loera-Valencia et al., 2019). Oppositely, high-fat diets raise the flow of oxysterols to the brain, which may impair BBB function (Petek et al., 2018). The metabolism of oxysterols usually involves CYP and studies showed that CYP46A1 and CYP27A1 and their metabolites were involved in AD pathology (Loera-Valencia et al., 2019). Under normal conditions, the human brain’s CYP46A1 levels increase significantly after birth and reach their equilibrium level within 1 year (Petrov and Pikuleva, 2019). In the early stages of AD, CYP46A1 levels decrease significantly while CYP27A1 levels increase. In the later stages of the disease, the oxysterols assessment showed a significant decrease in 24-hydroxycholesterol (24-OHC) content, while 27-OHC and 25-OHC increased significantly in the brains of AD patients (Petek et al., 2018). Thus, maintenance of cholesterol homeostasis is essential for consistent neuronal performance.

Besides, high levels of 27-OHC have been shown to activate the RAS in the brain, leading to oxidative stress, impaired cognitive function, and ischemic brain injury (Loera-Valencia et al., 2019). RAS is a cardiovascular regulatory system that, when dysregulated, triggers cardiovascular pathology, hypertension, oxidative stress, neuroinflammation, reduced cerebral blood flow, tissue remodeling, and disruption of memory consolidation and retrieval (Wright and Harding, 2019; Abiodun and Ola, 2020; Adhikari et al., 2021). There is also a local cerebral RAS that is indispensable in the pathophysiology of several neurodegenerative diseases (Vadhan and Speth, 2021). Some interactions between the brain and systemic RASs exist, in addition to the communication between the neuro-immunological processes and peripheral immune systems (Xue et al., 2020). Recent studies have found that in cognitively healthy older subjects, the use of angiotensin II receptor blockers (ARBs), but not brain angiotensin converting enzyme (ACE) inhibitors, was correlated with less amyloid deposition (Glodzik and Santisteban, 2021). Additionally, accumulating evidence suggests a convincing connection between RAS induced neuroinflammation and the onset of hypertension and dementia (Tran et al., 2022). Chronic RAS activation leads to considerable neuroinflammation which plays a crucial role in the pathogenesis of vascular dementia and AD (Cosarderelioglu et al., 2020). Synaptic dysfunction and neuronal cell death induced by neuroinflammation cause subsequent cognitive impairment (Tran et al., 2022). As mentioned earlier, the neuroprotective RAS pathway, regulated by angiotensin-converting enzyme 2 (ACE2) which converts angiotensin II into angiotensin-(1–7), can decrease both hypertension and dementia risk (Tran et al., 2022). The role and regulation of RAS with regard to neuronal function and its modulation remain to be characterized (Loera-Valencia et al., 2021). Strategies to reduce an overactive RAS might inhibit or improve cognitive impairment associated with hypertension and neuroinflammation (Wakayama et al., 2021). Future studies should address whether statins’ effect on memory can be attributed to RAS in regard to high levels of 27-OHC that can activate this system in the brain.

Besides, it has been suggested that the pleiotropic effects of statins stem from the decrease of the isoprenoid synthesis through the inhibition of the cholesterol synthesis pathway. However, this decrease has been suggested as a mechanism to reduce dementia risk in patients treated with statins. Indeed, the decrease of isoprenoids interferes with the intracellular migration of amyloid precursor proteins, resulting in reduced amyloid cleavage to Aβ. Thus, isoprenoid metabolism has been proposed as a target to develop new therapeutics for AD (Jeong et al., 2018).

### Anti-inflammatory effects of statins

Several studies have shown that statins have anti-inflammatory, antioxidant, and anti-thrombotic properties (Dai et al., 2021). Blocking the inflammatory signaling pathway increased *ApoE* expression in microglia, suggesting that *ApoE* levels and inflammation are in a negative feedback loop (Rebeck, 2017). A recent study, on iPSCs, investigated the functional and mechanistic consequences of *ApoE4* expression, such as the dysregulation of cholesterol metabolism in microglia and astrocytes, and elevated *de novo* cholesterol biosynthesis resulting from lysosomal sequestration of cholesterol (Julia et al., 2022). Aging has been shown to promote lipid metabolic dysregulation, inflammatory state, and increased AD risk, subsequent to neuronal debris accumulation of toxic protein aggregates in brain cells and decreased APOE-TREM2 (Triggering Receptor Expressed on Myeloid cells 2) in microglia (Julia et al., 2022). Additionally, the enriched matrisome includes increased inflammation, and lipid synthesis, and the proinflammatory state accelerates cholesterol biosynthesis but impairs efflux, suggesting *APOE4* changes matrisome signaling to exacerbate lipid dyshomeostasis (Julia et al., 2022). As the brain ages, a dysfunctional and proinflammatory state occurs due to microglia accumulating lipid-droplets (Marschallinger et al., 2020). Interestingly, *ApoE* expression increases in response to injury or stress and decreases when exposed to simvastatin *in vitro* using human astrocytes culture (Sodero and Barrantes, 2020). Additionally, statins may improve metabolic function and vascular health by reducing vascular inflammation and oxidative stress in patients with atrial fibrillation (Chinta et al., 2019). Statins can reduce the pro-inflammatory mediators, namely the tumor necrosis factor-alpha (TNF-α), Interleukin 1 beta (IL-1β), Prostaglandin E_2_ (PGE2), Interleukin-6 (IL-6), Interferon-gamma (IFN-γ), Cyclooxygenase-2 (COX-2), reactive oxygen species (ROS), and reactive nitrogen species (RNS) and induce the anti-inflammatory mediators such as Interleukin 10 (IL-10). Altogether, these results suggested that statins regulate the microglial activation and reduce neuroinflammatory mediators (Bagheri et al., 2020).

Furthermore, atorvastatin has been shown to reduce advanced glycation end products (AGEs), a set of modified proteins and lipids with harmful effects on neurodegeneration and Aβ formation. Atorvastatin can also suppress AGEs-induced expressions of nuclear factor kappa-light-chain-enhancer of activated B cells (NF-κB p65) and NADPH oxidase p47phox. The same study reported that atorvastatin attenuate AGEs-induced neuronal impairment by reducing inflammation and oxidative stress, through the inhibition of NADPH oxidase-NF-κB pathway (Li Z. et al., 2020). An animal study demonstrated that atorvastatin reverse memory dysfunction induced by inflammation (Solmaz et al., 2020). Besides, statins use in combination with conventional psychotropic medications had beneficial effects on cognition, presumably due to anti-inflammatory and anti-oxidant effects (Kim et al., 2019).

### The modulation of transcriptional activity by statins

The transcriptional signature and biological processes in AD brain may provide therapeutic targets for the disease (Johnson et al., 2020). In this regard, the development of AD is associated with Cerebral Atherosclerosis (CA) and its consequences. The molecular processes common to CA and AD were studied by Wingo et al. (2020) who identified 23 proteins associated with both AD and CA. CA was associated with increased oligodendrocyte development and myelination. However, no statistical interaction between CA and β-amyloid or tangles was found, suggesting that CA may contribute to AD independently of β-amyloid, and tau hallmarks. Hence these are additive risk factors of cognitive decline that can be studied according to their contribution to dementia (Wingo et al., 2020).

Statins may modulate the transcriptional activity of certain proteins in the brain which may be another mechanism for their beneficial effect on memory function. For instance, in terms of neuronal functions, statins can stimulate neurotrophin expression in brain cells by binding to Peroxisome Proliferator-Activated Receptor alpha (PPARα) and independently of the cholesterol synthesis pathway. Besides, simvastatin has been shown to enhance CREB (cAMP Response Element-Binding protein) and BDNF (Brain-Derived Neurotrophic Factor) in the hippocampus and consequently improves memory functions in mice (Roy et al., 2015). However, several studies reported that statins can worsen cognitive functioning by decreasing BDNF, irisin, NGF (Nerve Growth Factor), coenzyme Q10, or serotonin levels in the brain and conferring oxidative stress (Tan et al., 2019; Okudan and Belviranli, 2020; Deveau et al., 2021).

Furthermore, it has been shown that statins may increase the level of TGFBR2 (Transforming Growth Factor, Beta Receptor 2) and thus prevent the progression from MCI to AD (Fessel, 2020). Indeed, a decrease in TGFBR2 causes neuronal deficiency and might contribute to AD. Additionally, long-term lovastatin therapy has been shown to increase cell-surface levels of α7- and α4-nAChRs (nicotinic Acetylcholine Receptors). The stability of the nAChR is an important issue in cholinergic synapse function and AD pathology. Cholesterol levels regulate α7- and α4- nAChR levels differentially via dose-dependent mechanisms. Besides, α7-nAChR is essential in learning and memory function and is involved in AD pathology. Thus, by modifying cholesterol levels and α7-nAChR regulation, statin may exercise neuroprotective effects in rat hippocampal neurons (Borroni et al., 2020). Finally, activation of AMP-activated protein kinase (AMPK) is another possible underlying mechanism for the proposed neuroprotection of statins against Aβ-induced neurotoxicity. Statins can improve brain insulin action through the AMPK signaling pathway and exert protective effects on cognitive function (Jeong et al., 2018).

In conclusion, the precise mechanism of statin cognitive effects is still unclear. This might be attributed to the underlying mechanisms that were studied from different standpoints, in other words, different authors’ hypotheses based on pre-established results and different models used in each study might explain different suggested mechanisms. Moreover, as mentioned above, it has been shown that statins can have both cholesterol-dependent and –independent effects on cognition. For instance, the pleiotropic effects of statins are derived from the decline of the isoprenoid synthesis through the inhibition of the cholesterol synthesis pathway. Independent of cholesterol levels, statins have anti-inflammatory properties and can modulate the transcriptional activity of certain cerebral proteins with harmful effects on neurodegeneration.

## Discussion and concluding remarks

We have reviewed a broad range of studies examining statin-related cognitive effects including observational studies, randomized controlled trials, and case reports. We discussed the modifying factors that may alter the effects of statins on cognitive function and reported possible underlying mechanisms for these effects. We sorted these factors and discussed them individually and collectively.

One explanation for the inconsistent results of many studies on the cognitive benefits of statins is the heterogeneity of the study designs, namely, the variability of the cognitive tests and the small to medium sample sizes (Xuan et al., 2020). For instance, when the meta-analysis is conducted with small studies, the results are more diverse (Ying et al., 2021). The bias in the interpretations of studies’ results suggests a possible explanation for the inconsistency in mechanisms underlying the effects of statins on AD. The authors could investigate underlying mechanisms associated with specific outcomes study results, which may be either beneficial or detrimental from their specific perspectives. Additionally, the study models and experimental settings were different in each study, posing complications *per se*. For instance, statins have been shown to impair memory functions in rats by reducing levels of neurotrophins such as BDNF (Okudan and Belviranli, 2020), whereas the opposite effect has been reported in mice (Roy et al., 2015). Since statins can bind to and activate PPARα, which can enhance the expression of neurotrophins (Roy et al., 2015), it would be interesting to use human neurons-hippocampal tissue to clarify these effects.

Another explanation for the controversial role of statins in altering dementia risk lies in the underlying conditions of the patients. For example, TBI, concussion, depression, hypertension, and IHD should be considered when studying the cognitive effects of statins [as well as Body Mass Index (BMI), diabetes, angina, heart attack, hypertension, physical activity, sleep duration, smoking, and alcohol consumption habits] (Samaras et al., 2019; Alsehli et al., 2020). However, disorders associated with glymphatic pathway dysfunction, such as TBI, which is considered a risk factor for cognitive dysfunction (Viggiano et al., 2020), have not been included in study designs, not to mention the known genetic variants, sex, and dosage.

Previous studies showed that a main origin for reported dissimilarities in cholesterol level with AD incidence and the effects of cholesterol-lowering drugs across studies is the variable relationship between the time of cholesterol measurement or statin initiation and the time of onset and severity of AD (Shepardson et al., 2011a). Consistently, here we concluded that an important risk factor for memory loss and other diseases that influence cognitive function is aging (Nanna et al., 2018; Shetty et al., 2019; de Almeida et al., 2020; Orock et al., 2020; Viscogliosi et al., 2020). Statins have been shown to improve age-related conditions like CVD and AD (Chu et al., 2018; Newman et al., 2019; Shetty et al., 2019; van Dongen et al., 2019). However, the recent evaluation of the evidence for the long-term and short-term effects of statin therapy in patients with dementia older than 65 showed a small benefit in delaying the progression of AD (Davis et al., 2020). Moreover, the conflicting results between studies could be due to the wide age range of the patients (Alsehli et al., 2020).

Most importantly, as mentioned previously, each statin type has a different effect on cognitive function and demonstrates a different mechanism irrespective of lipophilicity. Thus, each statin type should be examined individually rather than as a group (i.e., lipophilic vs. hydrophilic drugs) or together. Overall, to better design a statistical model to study the statins’ cognitive effects, AD risk factors, the health profile of patients, genetic factors as well as the statin type, dose, and duration must be considered in the study design. This will eliminate the possible bias on study outcomes and control for confounding effects that could mask results or underlie causal factors.

Taken together, it is difficult to draw firm assumptions as human studies of statin effects have reported highly variable results with several confounding factors, including the AD stage at which statins were administered, different BBB permeability among statins, and the pleiotropic effects of statins, all of which provide the substantial variability observed (Shepardson et al., 2011b). Such factors, in addition to the new factors discussed here, could be considered in future assessments, perhaps in a categorical based manner, see Figure 1, as it is complicated for studies to take into account all confounding factors, including genetic variants, because it is expensive and laborious.

This work should be interpreted in the context of strengths and limitations. The lack of study design protocols to assess the cognitive effect of statins and the diversity of study designs did not allow us to conduct a more detailed review. There is not a common language with which researchers can generate and test hypotheses about the interactions among different dementia pathologic processes and syndromes (Jack et al., 2018). Correspondingly, some discrete data regarding evidently risk factors, such as higher BMI, smoking, alcohol abuse, and sedentary lifestyle, could not be easily established as a meta-analysis review. The major strength of our review is the detailed assessment of both individual-related and statin-related factors as well as possible underlying mechanisms of the effects of statins on cognitive performance, which allow researchers to develop a more robust study design.

In conclusion, the disagreements between studies on the effects of statins on cognitive function are likely related to the different modifying factors we have highlighted. The most hindering aspect of dementia management is its progression in later life, combined with other age-related conditions. Future studies should consider both individual-related and statin-related factors, to better scrutinize the influence of statins on dementia risk and cognitive performance. This will then inform future guidelines for clinicians to recognize potential beneficial factors and avoid detrimental factors that may alter the statin benefits on AD risk and cognitive impairment.

## Author contributions

TJ-T, SK, and MA-S wrote the draft manuscript. MG and HS performed the critical editing. All authors contributed to manuscript revision, read, and approved the final manuscript.
